# Hair Manganese and Hyperactive Behaviors: Pilot Study of School-Age Children Exposed through Tap Water

**DOI:** 10.1289/ehp.9504

**Published:** 2006-10-03

**Authors:** Maryse Bouchard, François Laforest, Louise Vandelac, David Bellinger, Donna Mergler

**Affiliations:** 1 Centre de recherche interdisciplinaire sur la biologie, la santé, la société et l’environnement (CINBIOSE), Université du Québec à Montréal, Montréal, Québec, Canada; 2 Department of Neurology, Children’s Hospital Boston, and Harvard Medical School, Boston, Massachusetts, USA

**Keywords:** children, CPRS-R, CTRS-R, hair, hyperactive behaviors, manganese, well water

## Abstract

**Background:**

Neurotoxic effects are known to occur with inhalation of manganese particulates, but very few data are available on exposure to Mn in water. We undertook a pilot study in a community in Québec (Canada) where naturally occurring high Mn levels were present in the public water system. Our objective was to test the hypothesis that greater exposure to Mn via drinking water would be reflected in higher Mn content in hair which, in turn, would be associated with increased level of hyperactive behaviors.

**Methods:**

Forty-six children participated in the study, 24 boys and 22 girls, 6–15 years of age (median, 11 years). Their homes received water from one of two wells (W) with different Mn concentrations: W1: mean 610 μg/L; W2: mean 160 μg/L. The Revised Conners’ Rating Scale for parents (CPRS-R) and for teachers (CTRS-R) were administered, providing T-scores on the following subscales: Oppositional, Hyperactivity, Cognitive Problems/Inattention, and ADHD Index.

**Results:**

Children whose houses were supplied by W1 had higher hair Mn (MnH) than those supplied by W2 (mean 6.2 ± 4.7 μg/g vs. 3.3 ± 3.0 μg/g, *p* = 0.025). MnH was significantly associated with T-scores on the CTRS-R Oppositional (*p* = 0.020) and Hyperactivity (*p* = 0.002) subscales, after adjustment for age, sex, and income. All children with Oppositional and Hyperactivity T-scores ≥ 65 had MnH > 3.0 μg/g.

**Conclusions:**

The findings of this pilot study are sufficiently compelling to warrant more extensive investigations into the risks of Mn exposure in drinking water.

Manganese is a naturally occurring element that constitutes approximately 0.1% of the earth’s crust, and low levels of Mn in water, food, and air are ubiquitous. In certain geologic regions, long contact times between ground water and bedrock enriched in Mn can lead to locally high levels in the water [U.S. Environmental Protection Agency (EPA) 2004]. Mn is an essential nutrient, and ingestion from drinking water is assumed to represent a small proportion of total intake, typically contributing < 1% although this can rise to 20% depending on Mn concentration in the water (Loranger and Zayed 1995). The rest of Mn intake occurs through food. Mn is thought to be better absorbed from water than from food, which is why the U.S. EPA proposed a modifying factor of three for assessing exposure to Mn from drinking water for the reference dose (RfD) (U.S. EPA 2004).

In certain conditions of exposure, Mn is a potent neurotoxicant (Mergler and Baldwin 1997). Our knowledge of the neurotoxic properties of Mn has emerged almost exclusively from inhalation exposure in the workplace, particularly in mining, ore crushing, ferro-Mn production, and welding. High exposure to airborne Mn has been associated with neurotoxic effects, with the worst cases displaying an extrapyramidal syndrome (manganism), characterized by gait dysfunction with a propensity to fall backward, postural instability, bradykinesia, rigidity, micrographia, masked facies, speech disturbances, and muscle tremors (Rodier 1955). Lower levels of exposure may produce neurobehavioral deficits involving motor and cognitive functions, as well as psychological perturbations (Iregren 1999; Levy and Nassetta 2003). The neuro-biochemical disturbances observed concurrently with clinical and subclinical effects of intoxication involve the striatal dopaminergic system, although indications of GABA (gamma-aminobutyric acid)-ergic and serotoninergic imbalance have also been reported (Dobson et al. 2004; Eriksson et al. 1987).

Only a few reports are available on the effects of exposure to Mn in water. A study conducted in Greece showed that adults > 50 years of age presented neurologic signs with increasing levels of Mn in drinking water (up to 2,000 μg/L) (Kondakis et al. 1989), but younger individuals were not included in this investigation. The reduced compensatory capacity of older persons’ nervous systems has been proposed as an explanatory mechanism for their apparent vulnerability (Kondakis et al. 1989). However, Vieregge et al. (1995) did not observe differences when comparing neurologic status and neurotoxic symptoms in adults > 50 years of age drinking water containing Mn concentrations 300–2,160 μg/L to those drinking water containing Mn < 50 μg/L. Small sample size, wide variation of Mn concentrations, and the use of extensive exclusion criteria that could have excluded persons presenting neurotoxic effects of Mn exposure (i.e., history of treatment for psychiatric disorder, neuroorthopedic incapacity for movement of hand and fingers) might have limited the validity of this study.

Two studies have been conducted on neurofunctional effects in children exposed to Mn through drinking water. The most complete investigation was conducted in the Chinese province of Shanxi, on 92 children, 11–13 years of age, exposed to 240–350 μg/L Mn in water; the contamination originated from sewage irrigation. Children from the contaminated area displayed lower performance on tests of manual dexterity and rapidity, short-term memory, and visual identification, when compared with children from a control area (He et al. 1994). Another study, conducted in Bangladesh with children 10 years of age, showed inverse relations between concentration of Mn in tube-wells and Full-Scale, Performance, and Verbal raw scores derived from tests drawn from the Wechsler Intelligence Scale for Children (WISC-III) (Wasserman et al. 2006). The levels of Mn in the wells varied widely, ranging from 4 to 3,908 μg/L (mean 795 μg/L), and arsenic was low (< 10 μg/L). In a case report of a child intoxicated with Mn from tap water, after well contamination, teachers had noticed his inattentiveness, and psychometric testing revealed markedly low verbal and visual memory (Woolf et al. 2002).

Several authors have hypothesized that excessive Mn could have detrimental effects on children’s behavior patterns. Indeed, some studies reported high hair Mn (MnH) levels in learning-disabled and hyperactive children when compared with controls (Barlow 1983; Collipp et al. 1983; Pihl and Parkes 1977). However, the meaning of these findings remains uncertain because of several methodologic problems, including choice of referent group (Barlow 1983), as well as possible confounding effects of other metals (Pihl and Parkes 1977). Furthermore, no Mn exposure source was identified, although Collipp et al. (1983) raised the hypothesis that it was infant formula with high Mn. The association between Mn exposure and children’s hyperactive behaviors remains plausible because the dopaminergic and GABAergic systems that play a role in hyperactivity in children (Li et al. 2006; Sagvolden et al. 2005) are also vulnerable to Mn (Fitsanakis et al. 2006).

In September 2005, we were informed that naturally occurring high Mn levels were present in the public water system of a small community in Québec and undertook a pilot study. Our hypothesis was that greater exposure to Mn via drinking water would be reflected in higher Mn content in hair which, in turn, would be associated with increased level of hyperactive behaviors.

## Materials and Methods

### Population and methods

The study was presented to the school principals and teachers of the primary and secondary schools of a small community (2,500 inhabitants), located 130 km north of Montreal, in Québec, Canada. The teachers who agreed to participate distributed a total of 175 recruiting letters to the children in their classes to bring home to their parents. The letter described the objectives of the study and stated that only families connected to the municipal aqueduct were eligible to participate. Forty-five families responded positively, but 15 were not connected to the municipal aqueduct and were excluded from the study sample; 13 responded negatively, and 119 did not respond at all. For this pilot study, we did not determine whether parents actually received the letters, nor did we follow-up the nonresponders; rather, we increased our sample through door-to-door visits on the streets where houses were connected to the public water system. A total of 47 children were enrolled in the study, but one child who refused to provide a hair sample was excluded.

The families were contacted by phone for an appointment. Each child provided assent, and signed informed consent was obtained from the parent. The study was approved by the Institutional Ethics Board of the Université du Québec à Montréal. Teachers were given Can$10 for each child evaluated.

### Data collection

#### Questionnaires and behavioral assessment

A questionnaire-based interview was conducted with one parent (80% mothers) and the child about sociodemographics, length of residence in the community, general health, and tap water uses (use for cooking, and amount ingested as water, in fruit juice made from concentrate and in soup). A dietary questionnaire was also administered, focusing on food rich in Mn (Wenlock et al. 1979). The frequency of consumption was surveyed for the following food or type of food: cereals, nuts, grains, brown rice, leafy vegetables, eggs, meat, dairy products, bananas, and canned fruit.

Behavioral ratings are considered efficient screening tools for attention deficit/hyperactive disorder (ADHD), a clinically heterogeneous disorder, involving inattention, hyperactivity, and impulsiveness (Brown et al. 2001). Given the exploratory nature of this study, we used only one instrument to assess children’s behavior; no other test was performed. We used the French version of the short form of the Revised Conners’ Teachers Rating Scale (CTRS-R) and Revised Conners’ Parents Rating Scale (CPRS-R) to assess ADHD behaviors (Conners 2000). These scales are widely used in Québec both in clinical and research practice with children and adolescents 3–17 years of age. They consist of 28 self-administered questions and take 5–10 min to complete. Four subscales are derived from the questionnaires: *a*) Oppositional (e.g., breaks rules, gets annoyed or angered), *b*) Hyperactivity (does not sit still, task persistence, restlessness, impulsivity), *c*) Cognitive Problems/Inattention (learns slowly, disorganized, cannot concentrate), and *d*) ADHD Index. The manual proposes that this index constitutes the best set of items for distinguishing children “at risk” for an ADHD diagnosis from children who are not.

The procedure described in the manual for handling instances of missing data was used on all subscales, except for the Cognitive/Inattention subscale of the CTRS-R. For this subscale, there were several unanswered items because the items refer to specific abilities (e.g., “Poor in arithmetic”), and the teacher who responded did not necessarily teach that subject matter. Thus, the score was not computed if there were more than two unanswered items; this was the case for five questionnaires. For each subscale, the score was transformed into a sex- and age-specific T-score based on a reference population composed of 1,897 children for the CTRS-R, and 2,426 children for the CPRS-R, from different regions in North America, including Québec. T-scores are standard scores, recalculated from raw scores so that each scale will have the same mean (50) and SD (10). According to the test manual, a T-score ≥ 65, which corresponds to the 93.3 percentile of the reference population, indicates “a significant problem” and should be a cause of concern (Conners 2000).

#### Hair sampling and analyses of Mn

A sample of scalp hair was taken in the occipital region, as close as possible to the skin. The 2 cm closest to the scalp were used for MnH analyses. The Québec Toxicology Centre of the Québec National Institute for Public Health (CTQ-INSPQ) performed the analyses. Hair samples were not washed before treatment and analysis. Samples were cut, weighed (~ 20 mg), and placed in Teflon digestion vessels (#026R; Savillex, Minnetonka, MN, USA). The hair samples were digested using concentrated nitric acid. The digestion vessels were placed in a ventilated oven at 110°C for 18 hr and then diluted to 10 mL with deionized water. The digested samples were analyzed by inductively coupled plasma-mass spectrometry (Elan 6000; PerkinElmer, Norwalk, CT, USA). Mn analyses were performed using an external calibration method and yttrium as the internal standard. The CTQ-INSPQ provides a normal reference range of 0–3 μg/g based on samples analyzed in Québec and a review article on hair reference intervals for trace elements (Miekeley et al. 1998). Here we used the upper level of this reference (3 μg/g) as a cut-off for elevated MnH.

Quality control measures included analysis of initial calibration verification standard, National Institute of Standards and Technology (Gaithersburg, MD, USA) Standard Reference Material (NIST-SRM) 1640 (trace elements in water), continuous calibration verification standards, procedural blanks, certified reference materials (CRMs) GBW 09101 and GBW 07601 (Human Hair, Shanghai Institute of Nuclear Research, Academia Sinica, China). Results were given as the average of three replicate measurements. The method detection limit was 0.01 μg/g. The average coefficient of variation over a 20-day period at a concentration of 2.6 μg/g was 4.4%.

#### Mn in the public water system

**Figure 1:**
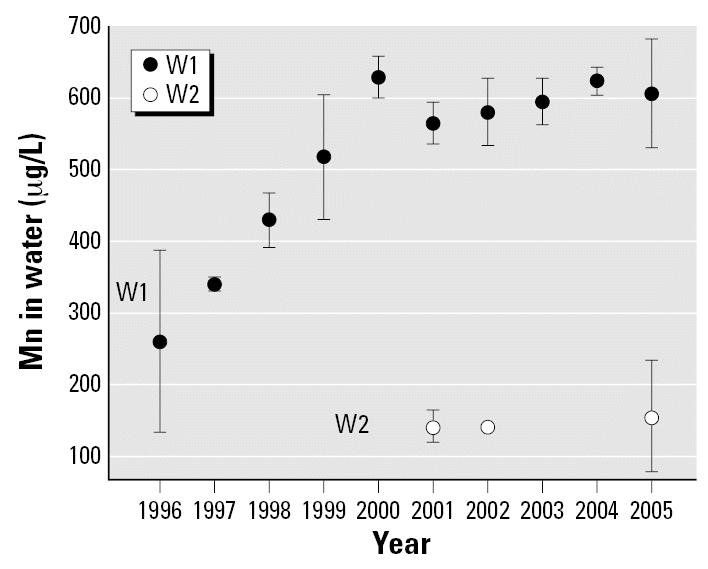
Evolution of Mn concentration [mean and 95% confidence interval (μg/L)] in the water of the public aqueduct system for the two wells (W1 and W2).

Most residences in this community are connected to the public water system, which takes its source in ground water. The aqueduct is supplied by wells located in two different sites. The municipality provided us with data on Mn levels over time. Mn content in water had been determined by argon flux plasma mass spectrophotometry (Optoma 4300DV; PerkinElmer, Boston, MA, USA). For the oldest well (W1), data were available for the past ten years (1996–2005). Over the years, Mn levels increased from 230 to 610 μg/L; the mean (± SD) level was 500 ± 129 μg/L. In 1999, a new well was drilled in a second site (W2); Mn levels in this well have been very stable and averaged 160 μg/L. The two wells supply water to different parts of the community. Figure 1 presents Mn concentration in water for the two wells. The house of each participating child was located on a map, allowing us to identify the corresponding well.

The bedrock in this region is characterized by high Mn content, and this is assumed to be the source of Mn in the ground water. There is no suspected anthropogenic source of Mn contamination in this low-industrialized area. The municipality has been aware of the high Mn levels in the water for several years, and was monitoring the situation, but at the time of the study no efficient water treatment system had been installed to remove Mn. There are negligible amounts of arsenic (< 0.002 mg/L), mercury (< 0.0001 mg/L), and lead (< 0.001 mg/L). Iron concentrations, however, follow a pattern similar to that of Mn and have been increasing since 1996; in 2005 the level was 2.37 mg/L, approximately 8 times the recommended level. Mn and iron concentrations are correlated (Pearson *r* = 0.747, *n* = 92). The water is treated by chlorination and sodium silicate to control the microbiologic parameters and remove particulates.

### Statistical analyses

We used SPSS version 13.0 (SPSS, Chicago, IL, USA), and the limit for statistical significance was set at *p* < 0.05. All significance testing was two-sided. The predictors of MnH levels were identified using regression modeling; the backward method was used, setting the *p*-value at 0.05 for a variable to enter, and at 0.1 to remove. The backward method is preferable to the forward method because it reduces type 2 error (e.g., missing predictor that does in fact predict the outcome) (Field 2005). Simple regressions were performed to assess the relations between potential covariates (age, sex, income) and T-scores on the subscales of the Conners’ scales for parents and teachers. Then we performed multiple regressions on MnH and T-scores after controlling for covariates. Interaction effects on T-scores were tested using the generalized linear model (GLM) procedure with sex, income, MnH (≤ or > 3 μg/g), and age (≤ or > 11 years). Finally, we performed chi-square tests to examine the distribution of elevated T-scores and high MnH levels, using the CTQ-INSPQ cut-off (> 3 μg/g). An elevated T-score was defined as ≥ 65, based on the test’s manual suggested threshold indicating a “significant problem” (Conners 2000). CTRS-R data were not available for two children, so the sample size was 44 for these analyses.

## Results

Forty-six children participated in the study, 24 boys and 22 girls. Age ranged from 6 to 15 years, with a median of 11.0 years. Ethnicity and language are homogeneous in this community, and likewise in the study sample: All 46 children were Caucasian and spoke French at home. Most children lived with two adults (78%). The socioeconomic status was high in the study sample, with only 20% of children living in households whose income was lower than the provincial average. A third of the children (30%) were born in the community, and 80% were ≤ 6 years of age when their family settled there. Table 1 presents the characteristics of the study population.

Twenty-eight children (61%) lived in houses connected to W1 (higher Mn) and 18 (39%) lived in houses connected to W2 (lower Mn). Most parents reported that they bought bottled water because of the bad taste of tap water. Thus, drinking tap water at home was very uncommon (9%), but tap water was used to prepare soups (59%) and fruit juice from concentrate (11%) and for cooking (96%). In contrast, most children drank tap water from the water dispensers at school (89%), which is supplied by W2, and several did so every day (78%). There was no difference in child’s age, sex, household income, or family structure between W1 and W2.

**Figure 2:**
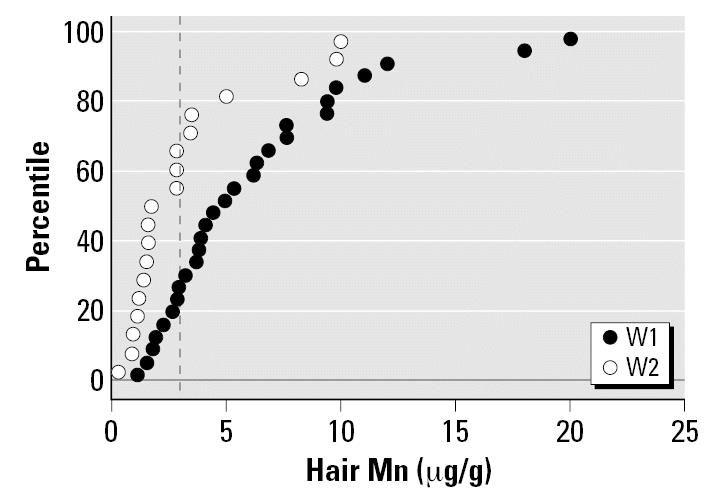
Distribution of MnH concentrations with respect to wells. Dashed line represents the upper reference limit for MnH.

MnH levels averaged 5.1 ± 4.3 μg/g, ranging from 0.28–20.0. Girls had significantly higher MnH than boys (mean 6.3 ± 4.4 μg/g vs. 4.0 ± 4.0 μg/g, *t*-test; *p* < 0.01), and those whose houses were serviced by W1 had higher levels (mean 6.2 ± 4.7 μg/g) than those serviced by W2 (mean 3.3 ± 3.0 μg/g; *t*-test, *p* = 0.025); Figure 2 shows the distribution of MnH levels with respect to well supply. Fifty-seven percent of the children had MnH levels > 3 μg/g, with W1 having a greater proportion (71%) than W2 (33%) (Fisher’s exact test, *p* = 0.016). The results of the multiple regression modeling showed that MnH was still significantly associated with sex (girls had more than boys; *p* = 0.012, partial correlation = 0.377), increasing age (*p* = 0.014, partial correlation = 0.368), and well (W1 had more than W2; *p* = 0.019, partial correlation = 0.353). These variables explained 30% of the variance. The different types of tap water use, the frequency of consumption of the different food types, duration of residency in the town, and age at arrival in the community did not enter into the model. Children born in the community had similar MnH levels to those of children who were not.

Parents reported behavioral problems serious enough to have consulted a specialist for nine children (20% of the group), although only two had received a diagnosis of ADHD and none was receiving methylphenidate or other ADHD medications. Children whose parents reported that their child presented behavioral problems had significantly higher T-scores than the others on all the CPRS-R subscales (Oppositional, *p* = 0.018; Hyperactivity, *p* < 0.000; and ADHD Index, *p* < 0.000), but on the CTRS-R this was the case only for T-scores on the ADHD Index subscale.

For the CTRS-R, simple regression analyses showed that MnH was significantly and positively associated with T-scores on the Oppositional (*p* = 0.005) and Hyperactivity (*p* = 0.001) subscales, and the relations with the two other subscales approached significance: Cognitive Problems/Inattention (*p* = 0.085) and ADHD Index (*p* = 0.062). There was no significant relation between MnH and T-scores on any CPRS-R subscale. Age was positively and significantly associated with higher T-scores for all subscales on the CTRS-R, but not the CPRS-R. Income was inversely and significantly associated with T-scores on several subscales for both parents’ and teachers’ ratings. Although there was no difference between girls and boys for the parents’ or teachers’ Conners’ scales, sex was retained as a covariate with age and income in all the analyses between MnH and Conners’ scales T-scores because girls had higher MnH than boys.

**Figure 3:**
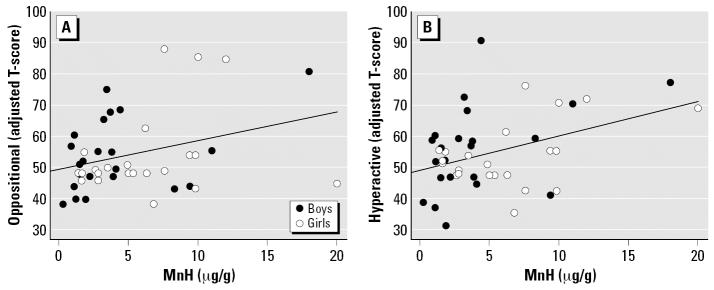
Adjusted T-scores (age, income) on the CTRS-R subscales as a function of levels of MnH (μg/g): (*A*) Oppositional (*y* = 49.0 + 0.937 × MnH, *R*^2^ = 0.106, *p* = 0.031, *n* = 44); (*B*) Hyperactivity (*y* = 49.1 + 1.103 × MnH, *R*^2^ = 0.156, *p* = 0.008, *n* = 44).

After controlling for the covariates, the relation between MnH and CTRS-R T-scores remained significant for both the Oppositional (*p* = 0.020) and Hyperactivity (*p* = 0.002) subscales (Figure 3). The two children with outlying MnH values (> 18 μg/g) were removed to determine their influence, and the resulting regression models were similar with no change in *p*-values. MnH levels were not significantly associated with T-scores for the Cognitive Problems/Inattention and ADHD Index subscales. Table 2 presents the results of the regression analyses between MnH and T-scores of CTRS-R subscales, before and after adjustment for age, sex, and income. For the CPRS-R, there was still no significant relation between MnH and T-scores even after inclusion of the covariates.

There was a significant interaction between age and MnH for the Hyperactivity subscale of the CTRS-R (GLM, *p* < 0.05). This interaction indicated that the positive relationship between MnH and Hyperactivity score was greater for older children (> 11 years) than for younger children. There was no interaction with sex or income.

The proportion of children with elevated T-scores (≥ 65) on the CTRS-R subscales was 18% for Oppositional, 23% for Cognitive Problems/Inattention, 20% for Hyperactivity, and 30% for ADHD Index; for the CPRS-R, the proportion was, respectively, 26%, 24%, 13%, and 22%. Children with high MnH levels (> 3 μg/g) were more likely to present elevated T-scores (≥ 65) on the CTRS-R, but not the CPRS-R, compared with those with lower MnH levels (Table 3). The differences were significant for three of the four subscales: Oppositional (*p* < 0.01), Hyperactivity (*p* < 0.01), and ADHD Index (*p* < 0.05). All of the children with elevated T-scores on the subscales Oppositional and Hyperactivity of the CTRS-R had MnH > 3 μg/g.

## Discussion

The present pilot study shows that children living in the houses connected to the well with higher Mn levels displayed higher concentrations of Mn in hair, pointing to tap water use as a source of Mn exposure in this community. In turn, MnH was associated with increased hyperactive and oppositional behaviors in the classroom. These relations remained significant after adjustment for income, age, and sex. It is notable that all of the children with elevated scores for CTRS-R Oppositional and Hyperactivity subscales had elevated MnH. Although bioindicators for other potential neurotoxicants were not assessed, data obtained from the municipality indicated that only negligible amounts of arsenic, lead, and mercury were present in the water supply, and the area has no major industries. Although iron deficiency might contribute to ADHD behaviors (Konofal et al. 2004), and although we did not assess iron status in children, iron levels were very high in the water supply.

The present study provides further support for MnH as a useful bioindicator for exposure to high Mn levels in water. Elevated MnH has also been observed in other community studies of exposure to Mn in water (He et al. 1994), and there are reports of dose-related variations in MnH with increasing Mn water content (Agusa et al. 2006; Kondakis et al. 1989). In the present study, MnH increased significantly with age and was higher in girls than in boys. In contrast, no age- or sex-related differences were reported in population-based studies of this element in the hair of adults (Rodushkin and Axelsson 2000) or children (Senofonte et al. 2000), although Khalique et al. (2005) reported that girls and women had higher MnH than men.

There have been several discussions about the usefulness of hair analysis and its standardization for studying Mn exposure [Agency for Toxic Substances and Disease Registry (ATSDR) 2001]. In the present study, the analytic protocol did not include washing the hair before determination of Mn content. We chose not to wash hair samples because substantial exterior contamination was not expected, unlike in occupational settings involving airborne particulates, and washing might have damaged the hair. Some argue that it is impossible to separate exterior from interior element content because elements from the exterior milieu might bond permanently in hair, whereas alteration of the content of certain elements might occur with time (ATSDR 2001). Factors that may influence Mn hair content include age of hair, color, and use of hair dye.

At the present time, we lack a well-defined reference value for MnH (ATSDR 2001) and, to date, bioindicators of Mn have not been included in the United States’ National Health and Nutrition Examination Survey (NHANES). We used 3 μg/g in MnH as an upper limit because it is the upper limit of the reference value provided by the laboratory that performs the toxicologic analyses for the Québec Institute of Public Health. It is a conservative upper limit because it was the highest of all of the studies reviewed by Miekeley et al. (1998) and > 2.41 μg/g, the maximum value reported in a study in Northern Sweden (Rodushkin and Axelsson 2000). The striking finding of MnH concentration > 3 μg/g for all children exhibiting elevated behavioral scores suggests that this concentration might have biologic significance.

Previous studies have reported effects of children’s exposure to Mn through water on IQ (Wasserman et al. 2006) and performance on several cognitive and neuromotor tests (He et al. 1994; Zhang et al. 1995). In the Chinese study, Mn exposure was associated with lower levels of dopamine, serotonin, norepinephrine, and acetylcholinesterase (Zhang et al. 1995). A recent pilot study in children residing near a hazardous waste site showed lower verbal memory and verbal IQ among those presenting both high arsenic and Mn levels in hair, but no association was observed between MnH and parents’ and teachers’ behavioral ratings (Wright et al. 2006). In the present pilot study, we focused solely on behavior and did not assess neuropsychologic functions.

There are several limitations to this pilot study. First, the small sample size leads to small statistical power, which nonetheless was sufficient to detect significant relationships. Second, the participants were self-selected and it is possible that parents who considered that their child had behavioral problems were more interested in volunteering; indeed, parents reported behavioral problems for 20% of the children. Furthermore, given the scope of the study, we did not collect information about nonrespondents. Third, a series of confounders were not evaluated—for example, maternal education, familial stress, and perinatal stress. Although we did not assess any other bioindicators of potential neurotoxicants, arsenic, lead, and mercury had been analyzed in the water supply and were found to be negligible. Among the strengths of the study are that the group was ethnically homogeneous, had an economic level above the provincial average, and most had a biparental family structure. Selection bias would not have influenced the teachers’ ratings, and parents had no knowledge of Mn concentration in their children’s hair.

The Mn content in the well with high Mn surpassed the World Health Organization (WHO) health-based water Mn guideline of 400 μg/L (WHO 2006). In Canada, the only current guideline for Mn concentration (50 μg/L) is based on aesthetic factors—staining and bad taste. The U.S. EPA recently issued a drinking water health advisory for Mn that yielded a lifetime health advisory value of 300 μg/L (U.S. EPA 2004). It is estimated that approximately 6% of private wells have Mn levels > 300 μg/L in the United States (Wasserman et al. 2006). In the present study, Mn levels in water were high; and although children reportedly did not drink much of it, those on the well with higher Mn had higher hair concentrations, suggesting that Mn exposure could occur through various pathways, such as in food cooked with water with a high Mn content. It has been proposed that showering could also contribute to Mn intake by inhalation of aerosols (Elsner and Spangler 2005).

The results of the pilot study are sufficiently compelling to warrant future studies on the effects of Mn in drinking water on behaviors in children. These studies should consider both behavioral and executive function outcomes because they provide complementary information. In addition, a series of covariates and confounders should be examined, such as family history of ADHD and other developmental problems, prenatal exposure to cigarette smoke, maternal education and intelligence, familial stress, perinatal stress, and exposure to other neurotoxicants (Hubbs-Tait et al. 2005). Finally, possible confounding by iron should be examined. Mn exerts a strong inhibitory effect on iron absorption (Boojar et al. 2002; Ellingsen et al. 2003), and iron deficiency per se is correlated with lower mental and motor test scores and with behavioral alterations in rats and humans (Sachdev et al. 2005).

## Conclusions

The findings of this pilot study indicate that exposure to high levels of Mn in tap water is associated with elevated MnH levels in children, and MnH is significantly associated with increased levels of hyperactive and oppositional behaviors in the classroom. The findings suggest a continuum in the relation between MnH and hyperactive behaviors in the classroom, with all children presenting elevated levels on certain scales having MnH levels above the normal range. A follow-up evaluation of these children is warranted to verify whether these behavioral problems persist once Mn concentrations are reduced. This pilot study lays the basis for a major study that would examine the relation between exposure to Mn in drinking water, MnH levels, and behavioral problems and neurofunctional deficits to establish adequate guidelines for drinking water.

## Figures and Tables

**Table 1:** Description of the study population and tap water use (*n* = 46).

Characteristic	Value
Age (years) [mean ± SD (range)]	11.0 ± 2.5 (6–15)
Percent male	52
Age when arrived in the community [mean ± SD (range)]	3.6 ± 3.6 (0–11)
MnH (μg/g) [mean ± SD (range)]	5.1 ± 4.3 (0.3–20.0)
Family structure	
Living with two adults (%)	78
Living with one adult (%)	22
Income < Can$40,000 (%)	22
Questionnaires respondent (% mother)	87
Behavioral problems reported by parents (%)	20
Drink tap water at home (%)	9
Use tap water for cooking at home (%)	96
Drink tap water at school (%)	89

**Table 2:** Regression models on T-scores of CTRS-R subscales and MnH, with and without covariates (age, sex, and income).

	Without covariates			With covariates			
CTRS-R subscales	B	SE	*p*-Value	B	SE	*p*-Value	Model R
Oppositional	1.299	0.434	0.005	1.172	0.483	0.020	0.500
Hyperactivity	1.587	0.427	0.001	1.478	0.443	0.002	0.638
Cognitive Problems/Inattention	0.952	0.538	0.085	0.244	0.488	0.620	0.666
ADHD Index	0.879	0.458	0.062	0.591	0.448	0.195	0.595

**Table 3:** T-scores (< or ≥ 65)^a^ on the CTRS-R subscales, stratified by MnH concentrations (≤ or > 3 μg/g).^b^

		MnH (μg/g)		
CTRS-R subscales	T-score	≤ 3	> 3	Fisher’s exact test *p*-Value
Oppositional (*n* = 44)	< 64	18	18	0.014
	≥ 65	0	8	
Hyperactivity (*n* = 44)	< 64	18	17	0.006
	≥ 65	0	9	
Cognitive Problems/Inattention (*n* = 39)	< 64	11	19	0.444
	≥ 65	5	4	
ADHD Index (*n* = 44)	< 64	16	15	0.043
	≥ 65	2	11	

a Cut-off suggested in the test manual (Conners 2000).

b Cut-off at the upper limit of the normal range (Miekeley et al. 1998).
